# *Dbx1*-Expressing Cells Are Necessary for the Survival of the Mammalian Anterior Neural and Craniofacial Structures

**DOI:** 10.1371/journal.pone.0019367

**Published:** 2011-04-28

**Authors:** Frédéric Causeret, Monica Ensini, Anne Teissier, Nicoletta Kessaris, William D. Richardson, Thibaut Lucas de Couville, Alessandra Pierani

**Affiliations:** 1 CNRS UMR 7592, Institut Jacques Monod, Univ Paris Diderot, Sorbonne Paris Cité, Paris, France; 2 Department of Cell and Developmental Biology and Wolfson Institute for Biomedical Research, University College London, London, United Kingdom; Instituto de Medicina Molecular, Portugal

## Abstract

Development of the vertebrate forebrain and craniofacial structures are intimately linked processes, the coordinated growth of these tissues being required to ensure normal head formation. In this study, we identify five small subsets of progenitors expressing the transcription factor *dbx1* in the cephalic region of developing mouse embryos at E8.5. Using genetic tracing we show that *dbx1*-expressing cells and their progeny have a modest contribution to the forebrain and face tissues. However, their genetic ablation triggers extensive and non cell-autonomous apoptosis as well as a decrease in proliferation in surrounding tissues, resulting in the progressive loss of most of the forebrain and frontonasal structures. Targeted ablation of the different subsets reveals that the very first *dbx1*-expressing progenitors are critically required for the survival of anterior neural tissues, the production and/or migration of cephalic neural crest cells and, ultimately, forebrain formation. In addition, we find that the other subsets, generated at slightly later stages, each play a specific function during head development and that their coordinated activity is required for accurate craniofacial morphogenesis. Our results demonstrate that *dbx1*-expressing cells have a unique function during head development, notably by controlling cell survival in a non cell-autonomous manner.

## Introduction

Building the vertebrate head relies on complex developmental programs involving molecular cross-talks between multiple cell types belonging to the three germ layers [1]. Within the developing brain, the acquisition of positional identity relies on the formation of morphogen gradients, whose combination leads to the nested expression of transcription factors, allowing sharp boundaries between compartments and morphological subdivisions of the developing brain to be formed [2]. Craniofacial structures develop in close proximity with the brain, and the same signalling molecules, namely Fgfs, Wnts, BMPs and Shh, are involved in both brain and face morphogenesis [3], [4]. However, patterning information is not exclusively mediated by morphogens passively diffusing from focal sources. For example, increasing evidence supports the hypothesis of alternative mechanisms involving the migration of signalling cells over long distances during cerebral cortex development [5].

Cephalic neural crest cells (CNCCs) are key players in vertebrate head development. They originate from the boundary between the surface ectoderm and neural plate and undergo epithelial-mesenchymal transformation to acquire a migratory phenotype. CNCCs generated at mesencephalic and diencephalic levels invade mainly the facial mesenchyme that will later give rise to the frontonasal structures, whereas CNCCs of rhombencephalic origin populate the branchial arches [6]. Genetic tracing experiments in mice [7] and chick-quail chimeras [8] demonstrated that most of the craniofacial tissues, including the entire facial skeleton, derive from CNCCs. In addition to their direct contribution, CNCCs were proposed to convey patterning information: in avian embryos, interspecies graft experiments demonstrated that CNCCs-derived signals regulate beak morphogenesis [9]. CNCCs were also shown to control brain patterning in chick embryos. Notably, ablation and electroporation experiments revealed that CNCCs migration into the facial mesenchyme and local secretion of BMP-antagonists is required to maintain *fgf8* expression in the forebrain [10], [11]. On top of their function in patterning neural tissues, CNCCs were proposed to control cell survival [12] as well as neural tube closure [13].

In mice, by contrast with chicks, little is known about the possible interactions between the cranial mesenchyme and the forebrain during development. In addition, evidence indicating that CNCCs are a source of signals involved in forebrain development is presently lacking.

Dbx1 is a homeodomain transcription factor previously shown to be expressed from E9.5 by restricted pools of progenitors in the central nervous system [14], [15]. Work performed in our laboratory indicated that, in the forebrain, cells deriving from these progenitors, born from E10.5 on, often share a migratory behaviour and a signalling function [16]–[18]. In this study, we identify five discrete subsets of *dbx1*-expressing cells in cephalic regions at early stages (E8.25–E8.75) of mouse development. We use a genetic ablation strategy to test the function of each of these subsets and reveal their relative involvement in forebrain and face development. We show by genetic tracing that the earliest *dbx1*-expressing cells generate a subpopulation of CNCCs which play a crucial role in head development by controlling proliferation and survival in neural and non-neural head tissues.

## Methods

### Animals

All animals used in this study were handled according to national regulations (approval #5096 from the French Ministry of Research on the use of genetically modified animals) and approved by the Veterinary Services of Paris (authorization to perform experimentation on vertebrate animals #75-1454). Genetic ablation of *dbx1*-expressing cells was achieved by conditional expression of the diphtheria toxin A fragment (DTA), crossing *Dbx1^IRES-loxP-Stop-loxP-DTA^* mice [16] (hereafter referred to as *Dbx1^DTA^*) with various Cre recombinase expressing strains. *PGK:Cre* animals [19] express Cre in the germ line, therefore allowing complete ablation of all *dbx1*-expressing cells. Targeted ablations of the different *dbx1*-expressing populations were achieved using the *Foxg1^Cre^* [20], *Wnt1:Cre* [21], *Nkx2.1:Cre* [22] and *Nestin:Cre* [23] lines. In order to perform genetic tracing of *dbx1*-expressing cells, we crossed *Dbx1^IRES-Cre^* [16] (referred to as *Dbx1^Cre^*) and *ROSA26^loxP-Stop-loxP-YFP^* [24] (referred to as *R26^YFP^*) mice. In *Dbx1^Cre^*;*R26^YFP^* embryos, Cre-mediated recombination occurs at the *ROSA26* locus in every cell expressing *dbx1*, leading to a permanent and irreversible expression of YFP.

Animals and embryos were genotyped by PCR using the appropriate specific primers and DNA extracted from the tail or yolk sack (for E8.5 and E9.5 embryos) as a template. At least three embryos of each genotype were analysed for each embryonic day.

### Embryo processing

For staging of the embryos, midday of the vaginal plug was considered as embryonic day 0.5 (E0.5). To stage E8.5 and E9.5 embryos more precisely, the somites were counted. Subtle variations in the size and morphology of embryos displaying the same number of somites were observed. Thus, we reasonably estimate that counting the somite for staging is accurate with a margin of error of 1ss. All embryos were collected and dissected in cold PBS and immediately fixed by immersion in 4% paraformaldehyde in 0.12 M phosphate buffer (PB) pH 7.2 at 4°C. Fixation duration was determined depending on the subsequent use of the embryos (see below). The neural tubes of E9.5 and E12.5 embryos were opened at the level of the rhombencephalon.

E8.5, E9.5 and E12.5 embryos subjected to immunostaining were fixed for 40–60 min, 60–90 min and 2 h, respectively. They were cryoprotected overnight in 10% sucrose in PB, embedded in a solution of 7.5% gelatine and 10% sucrose in PB, and frozen by immersion in isopentane cooled at −55°C. 20 µm thick cryostat sections were then obtained and collected on Superfrost Ultraplus slides (Menzell-Glasser).

Embryos used for *in situ* hybridisation, TUNEL or Nissl staining were fixed ≥4 h at 4°C. For storage, E8.5 and E9.5 specimens were dehydrated and stored in methanol whereas E12.5 heads were embedded in gelatine/sucrose.

### Immunostaining

The following primary antibodies were used: chick anti-GFP (AvesLab; 1∶2000), rabbit anti-AP2α (Santa Cruz; H-79; 1∶100), rabbit anti-PH3 (Upstate; 1∶500) and rabbit anti-activated Caspase-3 (Cell Signalling; 5A1; 1∶800). For fluorescent staining, secondary antibodies were purchased from Jackson Immunoresearch and slides were mounted in Vectashield with DAPI (Biovalley). In some cases, biotinylated secondary antibodies were used (Jackson Immunoresearch) and revealed using the Vectastain ABC kit (Vector) and diaminobenzidine (Sigma) as a substrate. Slides were then mounted in Mowiol.

### TUNEL

Whole mount TUNEL was performed using the Apoptag Peroxidase *in situ* Apoptosis Detection kit (Millipore) according to the manufacturer's instructions. Briefly, embryos were rehydrated and digested with 10 µg/mL proteinase K for 3 min. They were incubated with terminal deoxynucleotidyl transferase for 1 h at 37°C. The reaction was revealed using an alkaline phosphatase-conjugated anti-digoxigenin antibody (Roche) and NBT/BCIP (Roche) as a substrate.

### *In situ* Hybridisation

Digoxigenin-labelled riboprobes for *dbx1*, *dlx1*, *emx2*, *foxg1*, *ngn2*, *otx1*, *pax6*, *six3*, *sox10* and *wnt1* were synthesised with T3 or T7 RNA polymerases (Roche) using the Dig-RNA labelling mix (Roche) and 1 µg of linearised DNA as a template. *In situ* hybridisation on cryosections was performed as previously described [25]. For whole-mount staining, embryos were rehydrated, post-fixed in 4% paraformaldehyde, 0.2% glutaraldehyde in PBS, permeabilised in 1%SDS, 1%NP-40, 0.5% deoxycholate, 150 mM NaCl, 50 mM Tris pH 8, 1 mM EDTA and hybridised in a buffer containing 50% formamide, 5× SSC, 2% BBR, 2% SDS, 250 µg/mL yeast RNA, 100 µg/mL heparin and the probe (1∶50 dilution).

### Images acquisition

Embryos stained by whole-mount *in situ* hybridisation or TUNEL were incubated in 80% glycerol, 20% PBS and pictures acquired using a Zeiss Axiocam HRc color camera coupled to a Leica MZ FLIII stereomicroscope. Cryosections stained by Nissl, *in situ* hybridization or DAB immunohistochemistry were acquired using a Zeiss Axiocam HRc color camera coupled to a Zeiss Axiovert 200 microscope. Images of sections from E12.5 animals are composite that were generated by the Zeiss Axiovision software using Mosaix and Tiling functions to automatically reconstruct one image from multiple fields of the same specimen. Immunofluorescence stainings were acquired on a Leica TCS SP5 confocal microscope. All images of double-labelling correspond to a single confocal plane; in the case of sections stained with a single primary antibody, max projections of a 5–15 µm z-stack were used.

### Quantitative PCR

RNA preparation, reverse transcription and qPCR were performed as previously described [18]. Briefly, wild-type embryos were dissected in cold PBS. For 2ss and older embryos, only tissues anterior to the preotic sulcus (which marks the boundary between rhombomeres 2 and 3) were analysed. Several embryos of matching ages were pooled and RNA extraction was performed using the RNeasy Micro Kit (Qiagen) following the manufacturer's instructions. 100–300 ng of total RNA was used for cDNA synthesis using the SuperScript VILO cDNA synthesis kit (Invitrogen). Samples were prepared using an epMotion 5070 (Eppendorf) automated pipetting system and qPCR was performed on a LightCycler 480 (Roche) using the LightCycler 480 SYBR green Master I (Roche) reaction mix. *Dbx1* expression was calculated relative to the ribosomal protein S17 mRNA as previously described [18]. Experiments were performed at least in triplicates.

### Quantifications

Counting of the percentage of cells which are PH3^+^ in the neural plate and mesenchyme of E8.5 embryos was performed manually, using DAPI staining to identify individual cells. Only tissues located at presumptive midbrain and forebrain levels were taken into account. At least 1000 cells were counted for n = 3 control and 4 mutant embryos. The number of pyknotic nuclei per section was manually counted following thresholding of the DAPI staining to reveal the highest levels of fluorescence. Histograms represent mean ± sem. Significance was assessed using Student's t-test.

Counting of the percentage of Caspase-3^+^ cells which are YFP positive/negative was performed manually. Only Caspase-3^+^ cells which displayed a “normal” morphology (i. e. exhibiting a nuclear and a cytoplasmic compartment) were taken into account, thus excluding cell debris or cells that had reached advanced stages of cell death. The neuroepithelium was separated in two regions, one corresponding to the telencephalon (showing a high degree of recombination in *FoxG1^Cre^* embryos) and one corresponding to the mesencephalon and diencephalon (showing a high degree of recombination in *Wnt1:Cre* embryos). A total of >200 cells from two *FoxG1^Cre^;R26^YFP^;Dbx1^DTA^* embryos (at 12ss and 13ss) and three *Wnt1:Cre;R26^YFP^;Dbx1^DTA^* embryos (at 8ss and 10ss) were analysed.

## Results

### Genetic ablation of *dbx1*-expressing cells results in severe defects of forebrain and craniofacial structures

The Dbx1 transcription factor is expressed by restricted populations of progenitors during central nervous system development. In the forebrain, *dbx1* expression is notably detected at the septum, ventral pallium and preoptic area around E10.5–E12.5 [14]–[16]. In order to better understand the function of the various cell populations deriving from these progenitors, we have developed a genetic strategy to perform their conditional ablation. The use of the *Dbx1^loxP-stop-loxP-DTA^* mice [16] and the appropriate Cre recombinase-expressing strain enables us to selectively eliminate specific *dbx1*-expressing populations. We previously used *Nestin:Cre*, *Emx1:Cre*, *deltaNp73^Cre^* and *E1-Ngn2:Cre* mice to target Dbx1-derived subtypes generated between E10.5 and E12.5 [16]–[18]. Consistent with the limited number of *dbx1*-expressing cells, formation of the forebrain occurred in these mutants.

**Figure 1:**
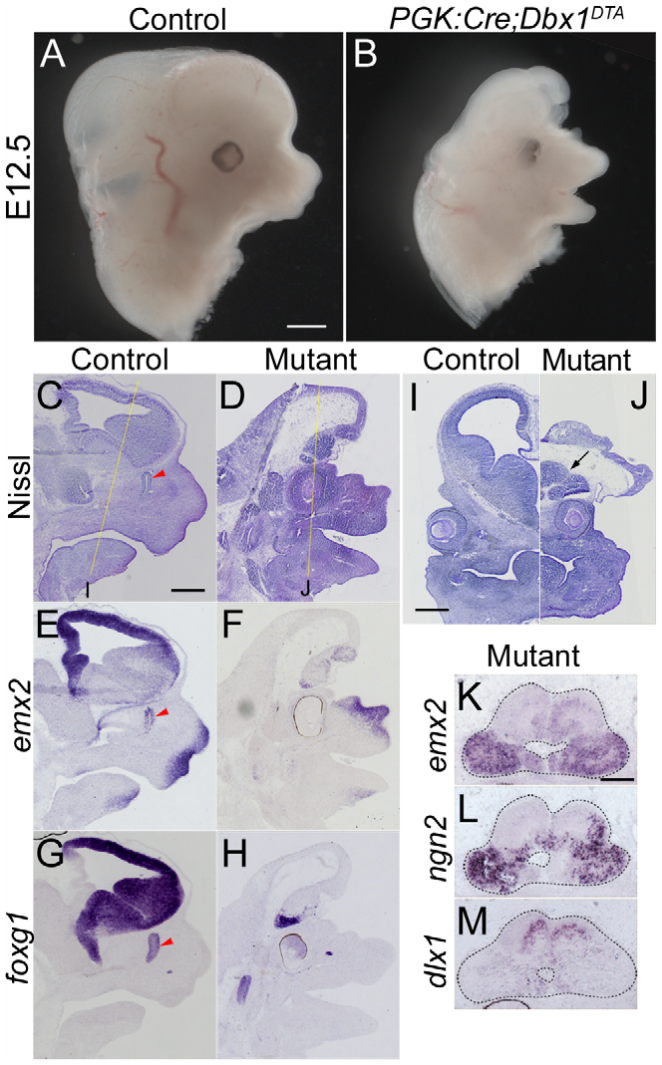
Genetic ablation of *dbx1*-expressing cells results in severe neural and craniofacial defects. Heads of wild-type (A) and *PGK:Cre;Dbx1^DTA^* (B) E12.5 mouse embryos. Mutants are exencephalic and display craniofacial malformations. (C–H) Alternate sagittal sections of wild-type (C, E, G) and *PGK:Cre;Dbx1^DTA^* (D, F, H) E12.5 embryos subjected to Nissl staining (C, D), *emx2* (E, F) and *foxg1* (G, H) *in situ* hybridisation. (I–J) Coronal sections of wild-type (I) and *PGK:Cre;Dbx1^DTA^* (J) E12.5 embryos stained by Nissl. The antero-posterior level at which coronal sections were collected is indicated by yellow dashed lines in C and D. The arrow in J points to the remaining telencephalon in ablated embryos. (K–M) High magnification of these telencephalic tissues in *PGK:Cre;Dbx1^DTA^* E12.5 embryo following *emx2* (K), *ngn2* (L) and *dlx1* (M) *in situ* hybridisation. Scale bars A: 500 µm, C, I: 400 µm, K: 200 µm.

In this study, we performed the complete ablation of all *dbx1*-expressing cells using the *PGK:Cre* line [19] in which recombination occurs ubiquitously at early stages (1–2 cells stage) of development. Surprisingly, severe craniofacial and brain morphogenesis defects were observed in E12.5 *PGK:Cre;Dbx1^DTA^* (Fig. 1A, B). Consistently, a similar phenotype has been observed using another ubiquitous and early Cre-expressing line [26] to ablate all *dbx1*-expressing cells (not shown).

Nissl staining performed on coronal and sagittal sections of E12.5 *PGK:Cre;Dbx1^DTA^* embryos indicated that the neural tube was opened at presumptive midbrain levels and aberrantly folded in rostral regions (Fig. 1C, D, I, J). Frontonasal structures were reduced in size and the nasal cavity was absent. The eyes, though shifted medially and incorrectly oriented, were present and their gross morphology conserved in ablated mutants (Fig. 1I, J). We performed i*n situ* hybridisation with *emx2* and *foxg1* riboprobes to label telencephalic structures and found that these territories were severely affected in the mutants, with only a small anterior territory being *emx2* and *foxg1* positive (Fig. 1E–H). The absence of olfactory epithelium, which normally expresses *emx2* and *foxg1*, was also confirmed in ablated mutants. The remaining telencephalic tissue observed in *PGK:Cre;Dbx1^DTA^* embryos appeared as a small vesicle (arrow in Fig. 1J) suggesting that neural tube closure did occur at the most anterior levels. Indeed, we observed that the *dlx1* ^+^ domain is relatively segregated from the *emx2* ^+^ and *ngn2* ^+^ domains (Fig. 1K–M), indicating that dorso-ventral patterning was maintained (ventral/*dlx1* ^+^ domain is found dorsal because of the folding of the remaining anterior nervous system; see Fig. 1D).

Taken together, these results indicate that the ablation of all *dbx1*-expressing cells results in an absence of dorsal neural tube closure at the midbrain level, a severe reduction in the size of the forebrain, as well as strong defects of craniofacial structures.

### Early *dbx1*-expressing cells are essential for forebrain and facial development

In a previous study, we performed the ablation of cells expressing the *dbx1* gene in the central nervous system starting from E10.5 by crossing *Nestin:Cre* and *Dbx1^loxP-stop-loxP-DTA^* animals [16]. E12.5 embryos resulting from such crosses displayed a much milder phenotype than *PGK:Cre;Dbx1^DTA^* mutants, consisting in an obvious reduction in the size of the mesencephalon and diencephalon (Fig. S1A, B). Importantly, despite the strong reduction in the thickness of the medial ganglionic eminence, the telencephalon of *Nestin:Cre;Dbx1^DTA^* appeared normal in size [16] (Fig. S1C, D) and mutants were undistinguishable from wild-type littermates with respect to craniofacial morphology (Fig. S1A, B). The major difference of phenotype between *Nestin:Cre* and *PGK:Cre* ablated mutants suggested that *dbx1*-expressing cells generated before E10.5 and/or outside of the central nervous system are essential for midbrain, forebrain and craniofacial development.

**Figure 2:**
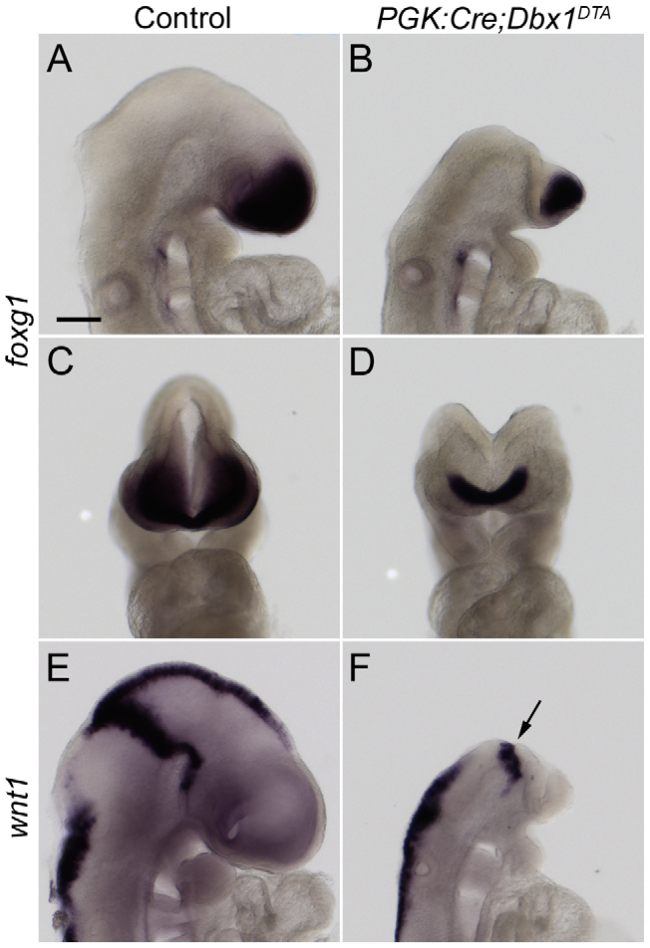
Loss of midbrain and forebrain tissues in ablated mutants. Side (A, B, E, F) and front (C, D) views of wild-type (A, C, E) and *PGK:Cre;Dbx1^DTA^* (B, D, F) E9.5 embryos (18–19ss) subjected to *foxg1* (A–D) and *wnt1* (E, F) whole mount *in situ* hybridisation. Most of neural tissues anterior to the midbrain-hindbrain boundary (arrow in F) are missing in ablated mutants. Scale bar: 200 µm.

We thus analysed earlier stages of development and found that *PGK:Cre;Dbx1^DTA^* embryos were already severely affected at E9.5. Dorsal midline closure failed to occur at midbrain and forebrain levels and *foxg1* expression was markedly reduced in ablated mutants, indicating that the telencephalic primordium was strongly reduced (Fig. 2A–D). Dorsal mesencephalic and posterior diencephalic territories, identified by *wnt1* expression, appeared missing whereas no obvious defects were observed in the rhombencephalon (Fig. 2E, F). At 10–12ss (E8.75) mutant embryos were already distinguished from wild-type littermates due to the failure in neural tube closure. Around 4–6ss (E8.5), the mutants could also be identified based on their morphology: measurements of the length of the headfold between the pre-otic sulcus and the anterior limit of the forebrain revealed a significant decrease in ablated mutants compared to wild-type embryos (21% reduction; Fig. S2B). We also measured a 14% reduction in the width of the neural plate at the mesencephalic level of 2–4ss (E8.25) embryos (Fig. S2A).

We thus find that morphological defects in ablated mutants are first seen at early somitic stages.

### Early *dbx1* expression pattern identifies several progenitor subsets

Our observation that ablated mutants display measurable defects from E8.25 were unexpected considering the initial report that *dbx1* expression only starts at E9.5 [15]. We therefore decided to carefully re-examine the *dbx1* expression pattern before E9.5 using *in situ* hybridisation.

**Figure 3:**
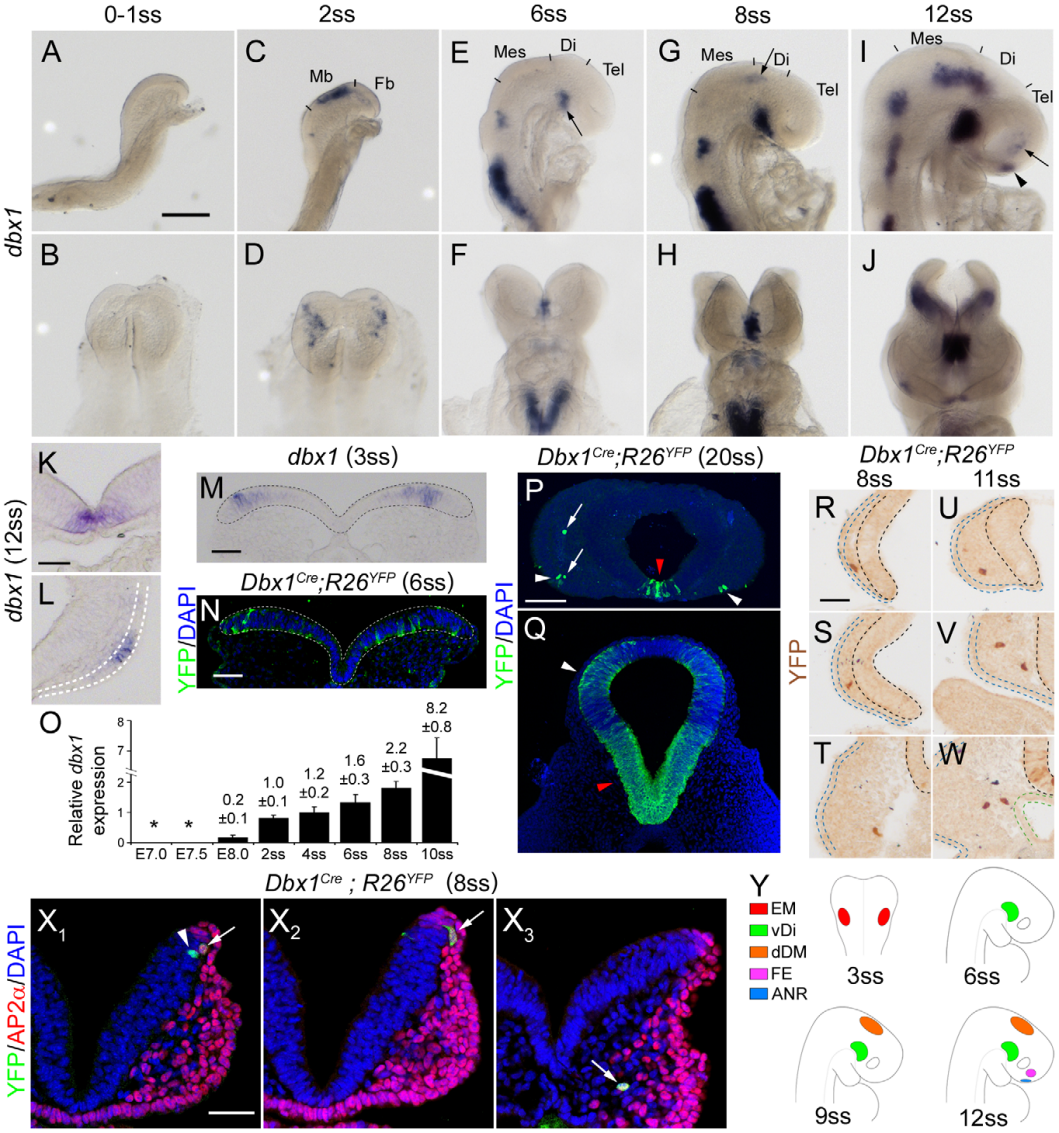
Dbx1 expression and tracing. (A–J) *Dbx1* expression analysed by whole-mount *in situ* hybridisation. B and D are dorsal views of the embryos presented in A and C respectively, rostral is up. F, H and J are frontal views of the embryos shown in E, G and I respectively. Antero-posterior subdivisions of the neural plate/tube are indicated. Mb: midbrain, Fb: Forebrain, Mes: mesencephalon, Di: diencephalon, Tel: telencephalon. Arrows in E, G and I point to the vDi, dDM and FE subsets respectively; the arrowhead in I shows the ANR subset. (K, L) Coronal sections through the forebrain of a 12ss embryo showing d*bx1* expression in the ANR (K) and facial ectoderm (L). (M) Coronal section collected at the midbrain level of a 3ss embryo illustrating *dbx1* mRNA expression; the neural plate is delineated by dashed lines. (N) Coronal section collected at the level of the midbrain of a 6ss *Dbx1^Cre^;R26^YFP^* embryo and stained for YFP (green) and DAPI (blue). (O) Quantification of *dbx1* expression by qPCR. Asterisks indicate stages at which *dbx1* could not be amplified. The relative expression values (normalised to 1 at 2ss and expressed in arbitrary units) are indicated on top of each bar as mean ± s.e.m. (P, Q) Coronal sections of a 20ss (E9.5) *Dbx1^Cre^;R26^YFP^* embryo at the level of the telencephalon (P) and di/mesencephalon (Q), stained for YFP (green) and DAPI (blue). YFP is detected in regions that express *dbx1* at earlier stages: ANR (red arrowhead in P), FE (white arrowheads in P), vDi and dDM (red and white arrowheads in P respectively) but also in cells located in the head mesenchyme (arrows in P). (R–W) Examples of YFP^+^ cells found in the head mesenchyme of *Dbx1^Cre^;R26^YFP^* embryos at 8ss (R–T) and 11ss (U–W) revealed by DAB immunostaining. Sections in R, S and U were collected at telencephalic levels, T, V and W at diencephalic levels. Neural tissues, surface ectoderm and endoderm are surrounded by black, blue and green dash lines respectively. (X) Coronal sections of a 8ss *Dbx1^Cre^;R26^YFP^* embryo immunostained for YFP (green) and the neural crest cells marker AP2α (red); cell nuclei are stained by DAPI (blue). Some of the YFP^+^ cells found in the neural plate are AP2α^−^ (arrowhead in X_1_) whereas YFP^+^ cells located at the most lateral aspect of the neural plate or in the mesenchyme (arrows in X_1_–X_3_) are AP2α^+^. Images X_1_–X_3_ correspond to single confocal planes. (Y) Schematic drawing of *dbx1* expression pattern. Scale bars: A: 200 µm; K, M, N, R, X: 50 µm; P: 100 µm.

With this technique, the earliest expression of *dbx1* mRNA we could detect was observed in 2ss embryos, in patches of cells located in the lateral/dorsal neural plate at the midbrain level (Fig. 3A–D, M). *Dbx1* expression at this location (subsequently referred to as EM, for “early midbrain”) persisted at 3ss and 4ss but was then downregulated by 6ss (Fig. 3E–F). At this stage *dbx1* expression started in the ventral diencephalon (vDi, arrow in Fig. 3E) and persisted later on (Fig. 3G–J). From 8ss (Fig. 3G–J), *dbx1* expression was found in the dorso-lateral diencephalon and mesencephalon (dDM, arrow in Fig. 3G) with this domain persisting and extending posteriorly at later stages (Fig. 3I). Of note, the dDM expression domain overlaps with the midbrain area in which *dbx1* is expressed at 2–4ss. Between 10ss and 15ss, *dbx1* expression was transiently found in the anterior neural ridge (ANR, arrowhead in Fig. 3I), the anterior limit of the forebrain, and in cells of the facial ectoderm (FE, arrow in Fig. 3I). Due to their small size, these two domains were best visualised on coronal sections (Fig. 3K, L). These results are summarised in Fig. 3Y.

We performed quantitative PCR (qPCR) experiments using RNA extracted from embryos at stages ranging from E7.0 to 10ss (E8.75). Consistent with *in situ* hybridisation experiments, we failed to detect *dbx1* mRNAs at E7.0 and E7.5 (Fig. 3O). *Dbx1* expression was detected at low levels in E8.0 extracts and markedly increased (by ∼5 fold) at 2ss (E8.25). Expression then subsequently increased ∼2 fold between 2ss and 8ss and ∼4 fold between 8ss and 10ss. These results therefore confirm the data obtained by *in situ* hybridisation.

We thus conclude that *dbx1* expression starts concomitantly to the formation of the first somites and is highly dynamic. In addition, we show for the first time that *dbx1* is expressed outside of the central nervous system.

### Tracing of early *dbx1*-expressing populations

Although telencephalic and frontonasal tissues are severely affected upon genetic ablation of all *dbx1*-expressing cells, we only found little expression of *dbx1* in theses structures. In order to test whether tissues that form the telencephalon and face derive from *dbx1*-expressing progenitors but no longer express *dbx1* mRNA, we used permanent tracing to follow the progeny of *dbx1*-expressing cells. We analysed *Dbx1^Cre^*;*R26^YFP^* embryos between E8.0 and E9.5 using YFP immunostaining to visualise cells deriving from *dbx1*-expressing progenitors. Consistent with expression data, we never observed YFP^+^ cells at E8.0. YFP expression was first detected in the mesencephalon at 6ss (Fig. 3N) with a pattern reminiscent of *dbx1* mRNA expression at 2–4ss (compare Fig. 3M and N). YFP^+^ cells were then found in the vDi around 9–10ss (shown at 11ss in Fig. S3B) and in the dDM around 12–13ss (Fig. S3F and data not shown). At E9.5 (20ss), YFP^+^ cells were detected in the ANR and FE (arrowheads in Fig. 3P) as well as in the vDi and dDM (Fig. 3Q). Thus, YFP expression at E8.5 and E9.5 in *Dbx1^Cre^*;*R26^YFP^* embryos recapitulates *dbx1* expression as observed by ISH. We found that the onset of YFP expression occurred ∼3–4ss later than that of the *dbx1* gene, indicating that the delay between mRNA expression and recombination and subsequent accumulation of the reporter protein spans around ∼5–6 hours (at this stage, one somite is formed every ∼90 minutes [27]).

One noticeable exception to the similarity between YFP and *dbx1* expression patterns was the presence of YFP^+^ cells in the head mesenchyme (arrows in Fig. 3P and Fig. 3R–W). Importantly, we never observed Dbx1 mRNA, protein, or LacZ expression in this tissue (in *dbx1^LacZ/+^* embryos, in which the *lacZ* gene has been inserted at the first ATG of the *dbx1* gene and allows to trace all Dbx1-derived cells without delay due to the recombination [25]). Mesenchymal Dbx1-derived cells were first found around 6–8ss, suggesting that they derive from the EM population (at this stage, only EM-derived cells are YFP^+^). At later stages, EM and dDM progenitor domains partially overlap, making it difficult to determine whether the dDM subset also contribute to these mesenchymal cells. Cephalic neural crest cells (CNCCs) are known to be generated at early somitic stages at the interface between the meso-diencephalic neural plate and surface ectoderm and to subsequently delaminate to invade the facial mesenchyme. We therefore tested the possibility that mesenchymal cells deriving from *dbx1*-expressing progenitors are CNCCs by performing double immunostaining for YFP and the transcription factor AP2α (a marker of NCCs [28]) on cryosections of *Dbx1^Cre^*;*R26^YFP^* embryos. We found that all YFP^+^ cells in the head mesenchyme, but also some in the lateral neural plate, were AP2α^+^ (Fig. 3X), showing that EM (and possibly dDM) *dbx1*-expressing progenitors generate CNCCs.

We therefore conclude that Dbx1-derived cells do not massively contribute to some of the territories that are most affected in ablated mutants, namely the forebrain and face, suggesting that the defects observed are non cell-autonomous.

### Genetic ablation of *dbx1*-expressing cells does not affect antero-posterior patterning of the neural plate but results in proliferation defects

**Figure 4:**
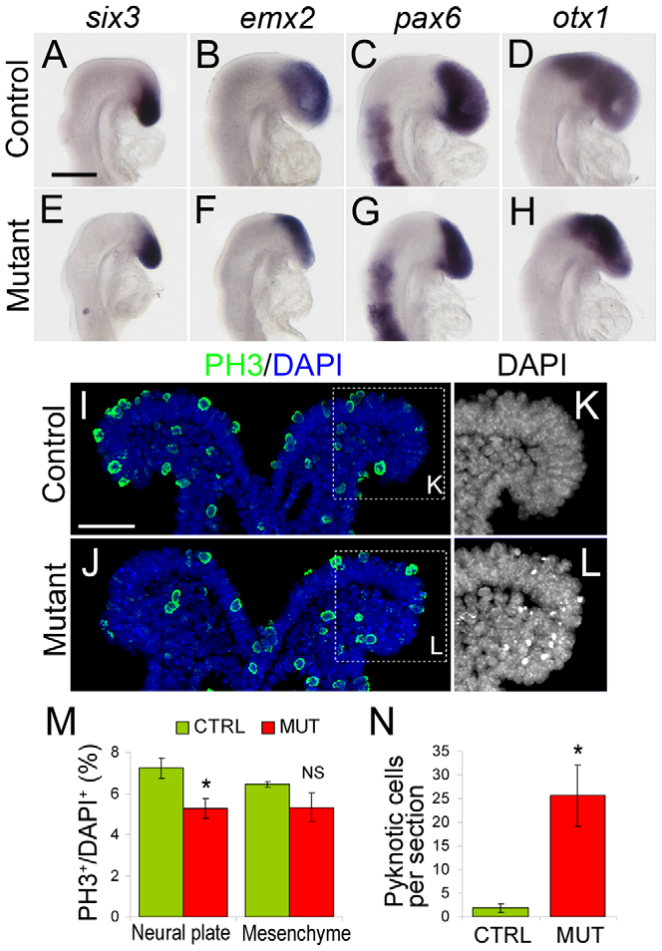
Antero-posterior regionalisation and proliferation in ablated mutants. (A–H) Whole-mount *in situ* hybridisation of 5 to 6ss control (A–D) and *PGK:Cre;Dbx1^DTA^* (E–H) embryos using probes against *six3* (A, E), *emx2* (B, F), *pax6* (C, G) and *otx1* (D, H). (I–J) Coronal sections collected at the level of the midbrain of 5ss control (I) and *PGK:Cre;Dbx1^DTA^* (J) embryos immunostained for phosphorylated histone-3 (PH3, green) and DAPI (blue). (K–L) Higher magnification of the DAPI staining, pyknotic nuclei are highly fluorescent. Quantifications (M, N) revealed a significant reduction in the number of PH3^+^ cells in the neural plate, but not in the mesenchyme, of mutant compared to controls (M) as well as a significant increase in the number of pyknotic cells per section (N). * p<0.05 in M and p<0.02 in N; n = 3 controls and 4 mutant embryos (4 to 8ss). Scale bars: A: 200 µm; I: 50 µm.

In order to determine the mechanisms that were responsible for the defects observed in *PGK:Cre;Dbx1^DTA^* embryos, we first tested whether the specification of forebrain tissues occurs correctly in ablated embryos. We analysed the expression of the neural regionalisation markers *emx2*, *otx1*, *pax6* and *six3* in *PGK:Cre;Dbx1^DTA^* and control embryos using *in situ* hybridisation. Analyses were done at 5–6ss, when the initial regionalisation of the neural plate in a hindbrain, midbrain and forebrain is achieved and when the ablated phenotype is already detectable. At this stage, in wild-type embryos, *six3* is expressed in the most anterior aspect of the neurectoderm (Fig. 4A), *emx2* identifies the dorsal telencephalic anlage (Fig. 4B), *pax6* expression is found in the forebrain and hindbrain (Fig. 4C), and *otx1* is expressed in the midbrain and forebrain (Fig. 4D). An identical expression pattern was observed in *PGK:Cre;Dbx1^DTA^* embryos, although the antero-posterior extent of the various domains appeared reduced compared to wild-types (Fig. 4E–H), consistent with the smaller size of mutants at this stage (see Fig. S2A, B). We therefore concluded that anterior neural structures are correctly induced and patterned in ablated mutants.

We also tested possible changes in the regulation of *dbx1* expression upon ablation of *dbx1*-expressing cells. At all stages analysed, we only detected very weak *dbx1* expression, which, most importantly, was systematically found in regions corresponding to *dbx1*-expressing areas in controls (Fig. S2C, 8D and data not shown), ruling out the possibility that the defects observed in ablated mutant result from ectopic upregulation of *dbx1* (and hence, ectopic toxin production).

We then analysed proliferation using phospho-histone 3 (PH3) immunostaining on 4 to 8ss embryos. Quantifications revealed a mild (27%) but significant decrease in the percentage of PH3^+^ (i. e. mitotic) cells in the neural plate of ablated mutants compared to controls (Fig. 4I, J, M). By contrast, we found no significant differences in the percentage of PH3^+^ cells in the head mesenchyme. It therefore appears that proliferation in the neural tube is reduced upon ablation of *dbx1*-expressing cells.

Interestingly, careful examination of DAPI staining on these specimens revealed a ∼10 fold increase in the number of pyknotic nuclei compared to control littermates (Fig. 4K, L, N), suggesting enhanced cell death occurred in ablated embryos.

### Genetic ablation of *dbx1*-expressing cells triggers extensive apoptosis

**Figure 5:**
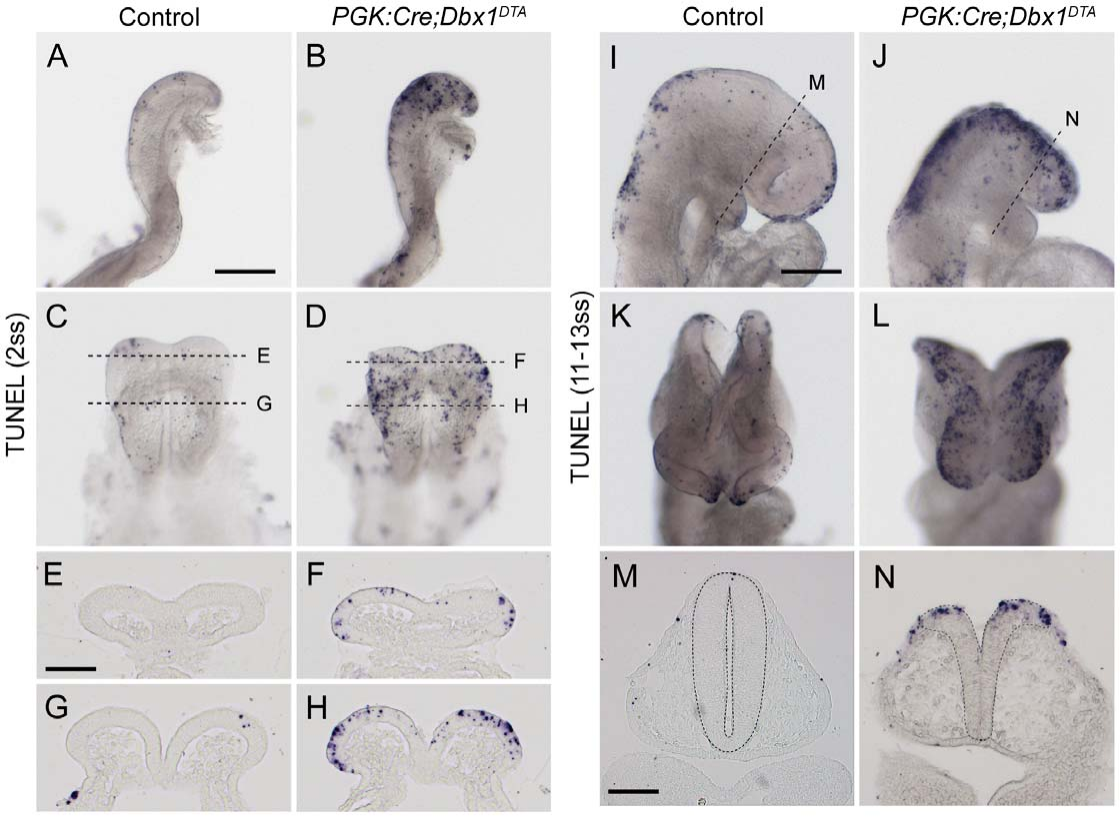
Increased apoptosis in ablated mutants. Whole-mount TUNEL staining of 2ss (A–D) and 11–13ss (I–L) control (A, C, I, K) and *PGK:Cre;Dbx1^DTA^* (B, D, J, L) embryos. C and D are dorsal views of the embryos shown in A and B respectively. (E–H) are cryosections of the embryos shown in A and B at the level of the forebrain (E, F) and midbrain (G, H) as indicated by dashed lines in C and D. K and L are front views of the embryos shown in I and J respectively. M and N are cryosections collected at the level of the diencephalon of these embryos as indicated by dashed lines in I and J. Neural tissues are surrounded by a dashed line for better visualisation. Scale bars: A, I: 200 µm; E, M: 100 µm.

To test whether tissues were undergoing apoptosis in ablated mutants, we performed whole-mount TUNEL staining. At E8.0, consistent with the fact that *dbx1* expression is just starting (not yet detected by *in situ* hybridisation but found at low levels by qPCR), both ablated and wild-type littermates displayed low levels of apoptosis (Fig. S2D). At 2ss, increased apoptosis was found throughout the neural plate (Fig. 5A–D), in the forebrain (Fig. 5E, F) and midbrain (Fig. 5G, H), but not at spinal cord levels (Fig. S2E). Of note, TUNEL staining was typically more pronounced in the lateral aspect of the neural plate and at the junction with surface ectoderm. At later stages (11–13ss), increased TUNEL-staining was also observed in ablated mutants compared to wild-types (Fig. 5I–L). Reminiscent of earlier stages, apoptotic cells were mainly located in the dorsal and anterior regions of the neural tube as well as in the surface ectoderm, especially at the level of the forebrain (Fig. 5M, N).

Importantly, we found striking differences between the pattern of *dbx1* expression in wild type embryos and the distribution of apoptotic cells in ablated animals. At 2–4ss, when *dbx1* is expressed in a patch of cells in the midbrain (EM subset), ablated mutants of the same stage display apoptosis throughout the neural plate, including the most anterior and lateral regions (compare Fig. 3D and 5D). At 10–12ss, when four distinct *dbx1*-expressing populations are identified in control embryos (vDi, dDM, ANR and FE), *PGK:Cre;Dbx1^DTA^* littermates exhibit a continuous stripe of TUNEL^+^ cells in the dorsal midbrain and forebrain (compare Fig. 3I and 5J). Furthermore, the number of apoptotic cells observed in the telencephalon of *PGK:Cre;Dbx1^DTA^* at 11–18ss (Fig. 5J, L and data not shown) by far exceeded the one of Dbx1-derived cells as indicated by genetic tracing in 20ss *Dbx1^Cre;^R26^YFP^* embryos (Fig. 3P).

Together, these results indicate that the apoptosis observed in ablated mutants starts almost concomitantly to the onset of *dbx1* expression and, in a large extent, occurs in regions that neither express *dbx1* nor derive from Dbx1-expressing progenitors. They also suggest that *dbx1*-expressing cells present at 2–4ss promote the survival of neighbouring tissues in a non cell-autonomous manner.

### Specific requirements of the *dbx1*-expressing subpopulations for forebrain and head development

In order to directly test the function of the different *dbx1*-expressing subsets in forebrain and head development, we attempted to perform their targeted ablation. To this end, we used *Nkx2.1:Cre* [22], *Wnt1:Cre* [21] and *Foxg1^Cre^* [20] mouse lines that we crossed with *Dbx1^loxP-stop-loxP-DTA^* animals.

We first analysed the pattern of recombination by crossing these Cre-expressing mice with *ROSA26^loxP-stop-loxP-YFP^* reporters and compared it to the distribution of Dbx1-derived cells at E8.5. The *Nkx2.1:Cre* line allowed recombination in a domain encompassing the vDi *dbx1*-expressing population (Fig. S3A, B). We confirmed effective ablation of this subset in *Nkx2.1:Cre;Dbx1^DTA^* embryos by analysing *dbx1* expression at E9.5 (19ss; Fig. S3C, D). With the *Wnt1:Cre* line, recombination occurred in the dorsal mesencephalon and diencephalon, overlapping with the dDM *dbx1*-expressing population (Fig. S3E, F). *Dbx1* expression at E9.5 (19ss) in *Wnt1:Cre;Dbx1^DTA^* embryo indicated a complete ablation of the dDM subset (Fig. S3G, H). The *Foxg1^Cre^* line drove recombination in the most anterior part of the neural tube as well as the surface ectoderm at stages preceding the onset of *dbx1* expression in the ANR and FE (Fig. S3I). *Dbx1 in situ* hybridisation on *Foxg1^Cre^;Dbx1^DTA^* 14ss embryos confirmed the efficient ablation of both the ANR and FE subsets (Fig. S3O–T). In addition, the *Foxg1^Cre^* line allowed a salt-and-pepper recombination in the neural tube and mesenchyme all along the antero-posterior axis (Fig. S3J), suggesting that at least some *dbx1*-expressing cells from the vDi and dDM subsets are also targeted. However, the extent of such an ectopic recombination did not appear to significantly affect these populations as revealed by *dbx1 in situ* hybridisation on *Foxg1^Cre^;Dbx1^DTA^* E9.5 (20ss) embryos (Fig. S3K, L).

At last, we found that in both the *Foxg1^Cre^* and *Wnt1:Cre* lines, some recombination occurs at early somitic stages in the area of *dbx1* expression in the midbrain (Fig. S3M, N; compare with Fig. 3N), suggesting that the EM subset is partially targeted by these two lines. It is also worth noting that CNCCs in the head mesenchyme were targeted by the *Wnt1:Cre* line (Fig. S3E, N; as previously described [21]).

In summary, the *Nkx2.1:Cre* line targets the vDi subset, the *Foxg1^Cre^* line partially targets the EM, vDi and dDM subsets and completely targets the ANR and FE subsets, and the *Wnt1:Cre* line partially targets the EM subsets and completely targets the dDM subset (Table 1).

**Table 1:** Extent by which each *dbx1*-expressing subset is targeted by Cre-mediated recombination using the different mouse lines.

Stage	2–4ss	6ss→	8ss→	10–15ss	
Population	EM	vDi	dDM	ANR	FE
***Nkx2.1:Cre***	-	Complete	-	-	-
***Foxg1^Cre^***	Partial	Partial	Partial	Complete	Complete
***Wnt1:Cre***	Partial	-	Complete	-	-
***Triple-Cre***	Partial	Complete	Complete	Complete	Complete
***PGK:Cre***	Complete	Complete	Complete	Complete	Complete

**Figure 6:**
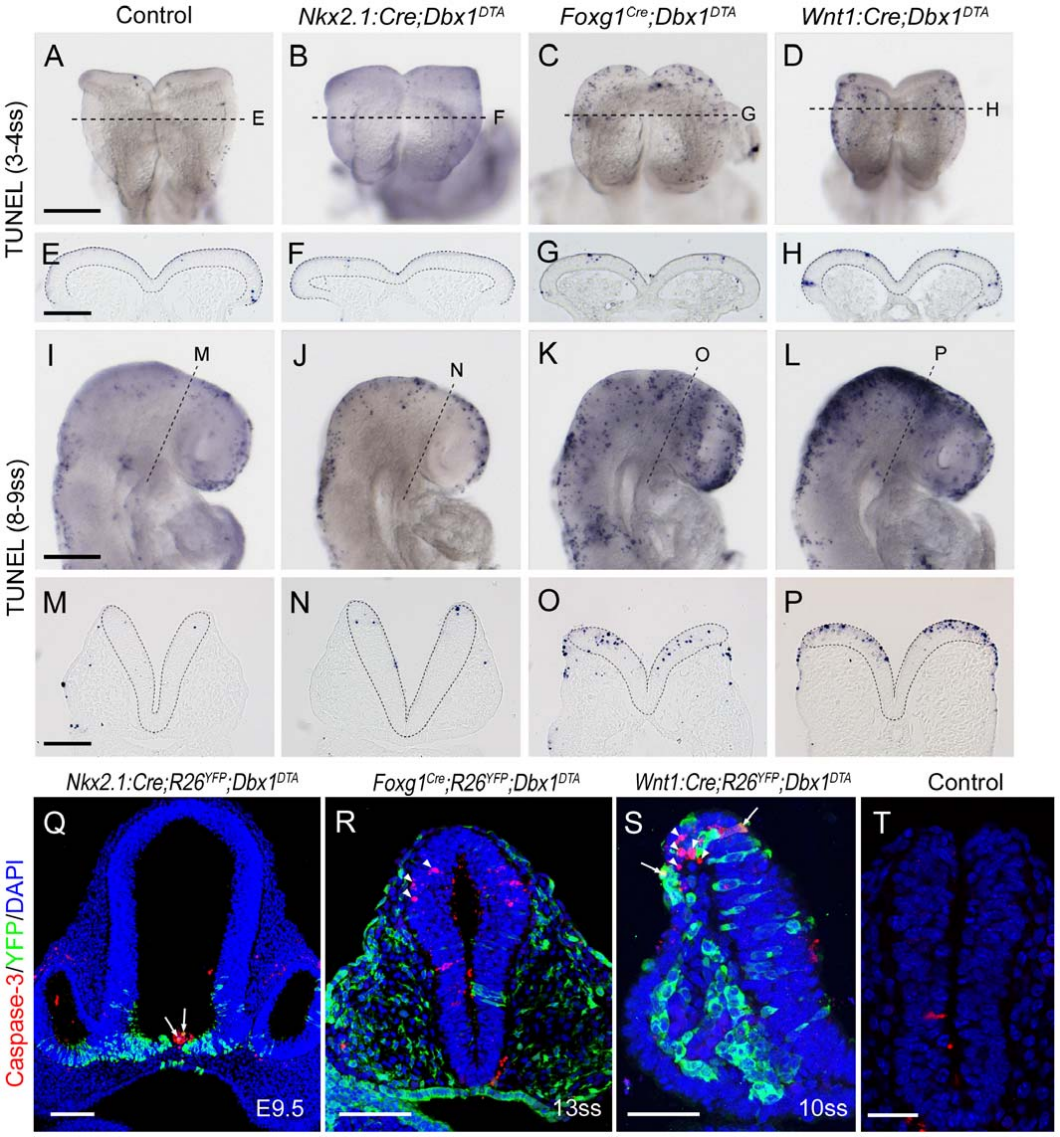
Apoptosis upon targeted ablations. Whole-mount TUNEL staining of 3 to 4ss (A–D) and 8 to 9ss (I–L) control (A, I), *Nkx2.1:Cre;Dbx1^DTA^* (B, J), *Foxg1^Cre^;Dbx1^DTA^* (C, K) and *Wnt1:Cre;Dbx1^DTA^* (D, L) embryos. E–H and M–P are cryosections of the embryos shown in A–D and I–L collected at the level indicated by a dashed line. Neural tissues are surrounded by a dashed line for better visualisation. (Q–T) Immunostaining for activated Caspase-3 (red), YFP (green) and DAPI (blue) on coronal cryosections of *Nkx2.1:Cre;R26^YFP^;Dbx1^DTA^* (Q), *Foxg1^Cre^;R26^YFP^;Dbx1^DTA^* (R), *Wnt1:Cre;R26^YFP^;Dbx1^DTA^* (S) and control (T) embryos. Images in Q–T correspond to a single confocal plane. Arrows point to Caspase-3^+^/YFP^+^ cells, arrowheads indicate Caspase-3^+^/YFP^−^ cells. Scale bars: A, I: 200 µm; E, M, Q, R: 100 µm; S, T: 50 µm.

We then analysed apoptosis in the various selectively ablated mutants. Interestingly, *Nkx2.1:Cre;Dbx1^DTA^* embryos displayed the same low levels of apoptosis than their wild-type counterparts between 3ss and 15ss (Fig. 6A, B, E, F, I, J, M, N and data not shown). We therefore concluded that the vDi *dbx1*-expressing population is dispensable for the survival of anterior tissues. Both *Foxg1^Cre^;Dbx1^DTA^* embryos (Fig. 6C, G) and *Wnt1:Cre;Dbx1^DTA^* (Fig. 6D, H) showed increased apoptosis from 3–4ss compared to controls (Fig. 6A, E), but in an extent that did not match the levels observed in *PGK:Cre;Dbx1^DTA^* mutants (Fig. 5D, H). Such an observation not only confirms that the EM subset is essential for the survival of the neural plate cells, but also that it is partially (but not completely) targeted by the *Wnt1:Cre* and *Foxg1^Cre^* lines. At 8–9ss, increased apoptosis was observed in *Wnt1:Cre;Dbx1^DTA^* and *Foxg1^Cre^;Dbx1^DTA^* embryos (Fig. 6I, K–M, O, P). With the *Foxg1^Cre^* line apoptotic cells were typically sparse along the antero-posterior and dorso-ventral axis of the forebrain and midbrain; many were also found in the facial surface ectoderm (Fig. 6K, O). By contrast, using the *Wnt1:Cre* line, increased apoptosis was most prominent in the dorsal midbrain (Fig. 6L, P).

Each targeted ablation therefore yields a specific pattern of apoptosis, and none of them recapitulates the complete ablation.

In order to precisely determine which cells undergo apoptosis in the different ablated embryos, we generated mice bearing both the *Dbx1^loxP-stop-loxP-DTA^* and the *ROSA26^loxP-stop-loxP-YFP^* alleles and mated them with Cre-expressing lines. We performed immunostaining for activated Caspase-3 and YFP on embryos resulting from such crosses to reveal on the same specimen apoptotic and recombined cells respectively. When using the *Nkx2.1:Cre* line, we found Caspase-3^+^ cells in the vDi, with most of them also being YFP^+^ (arrows in Fig. 6Q), showing that these cells died because they were located in the recombination domain and expressed *dbx1* (and therefore DTA). When recombination was driven using the *Foxg1^Cre^* strain, we observed increased apoptosis (similar to TUNEL staining) and, interestingly, we counted that 163 out of 185 Caspase-3^+^ cells found in the mesencephalon and diencephalon were YFP^−^ (i. e. not recombined; arrowheads in Fig. 6R and Fig. S4). Similarly, with the *Wnt1:Cre* line, we counted that 69 out of 81 apoptotic cells found in the forebrain region were not recombined (arrowheads in Fig. 6S and Fig. S4), ruling out the possibility that they died because of DTA expression. Control embryos that did not express Cre recombinase displayed very low levels of Caspase-3 staining (Fig. 6T). Taken together, these data show that a significant part of the apoptosis observed in ablated mutants is non cell-autonomous as previously suggested from the analysis of *PGK:Cre;Dbx1^DTA^* embryos.

In addition to cell survival defects, we found that *Wnt1:Cre* ablated embryos display a number of forebrain patterning defects. Thus, around 8ss, we observed a dorsal and medial expansion of the *foxg1* expression domain (Fig. S5A–D) associated with a posterior shift in the dorsal limit of the *emx2* expression domain (Fig. S5E–H). Since the *Wnt1:Cre* line does not drive recombination in the forebrain, we believe that these defects are strong evidences in favour of a non cell-autonomous function of the dorsal midbrain on forebrain patterning.

### Early midbrain *dbx1*-expressing cells play a crucial role in forebrain formation

**Figure 7:**
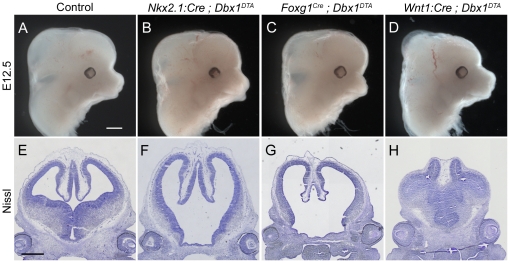
Consequences of targeted ablations at E12.5. Heads of wild-type (A), *Nkx2.1:Cre;Dbx1^DTA^* (B), *Foxg1^Cre^;Dbx1^DTA^* (C) and *Wnt1:Cre;Dbx1^DTA^* (D) E12.5 mouse embryos. (E–H) Nissl staining of coronal sections collected at the level of the eyes of the embryos shown in A–D. Scale bars: 500 µm.

When we examined the outcome of targeted ablations at E12.5, we found that each mutant exhibited a mild facial phenotype (Fig. 7A–D). Importantly, all three single mutants formed a forebrain that was near normal in size, although its patterning was affected (Fig. 7E–H and data not shown). Thus, each targeted ablation results in a specific phenotype and none of them recapitulate the complete ablation. This could either be explained by a cooperative activity of different subsets, or by the fact that the EM subset is not completely ablated in any of the single mutants.

**Figure 8:**
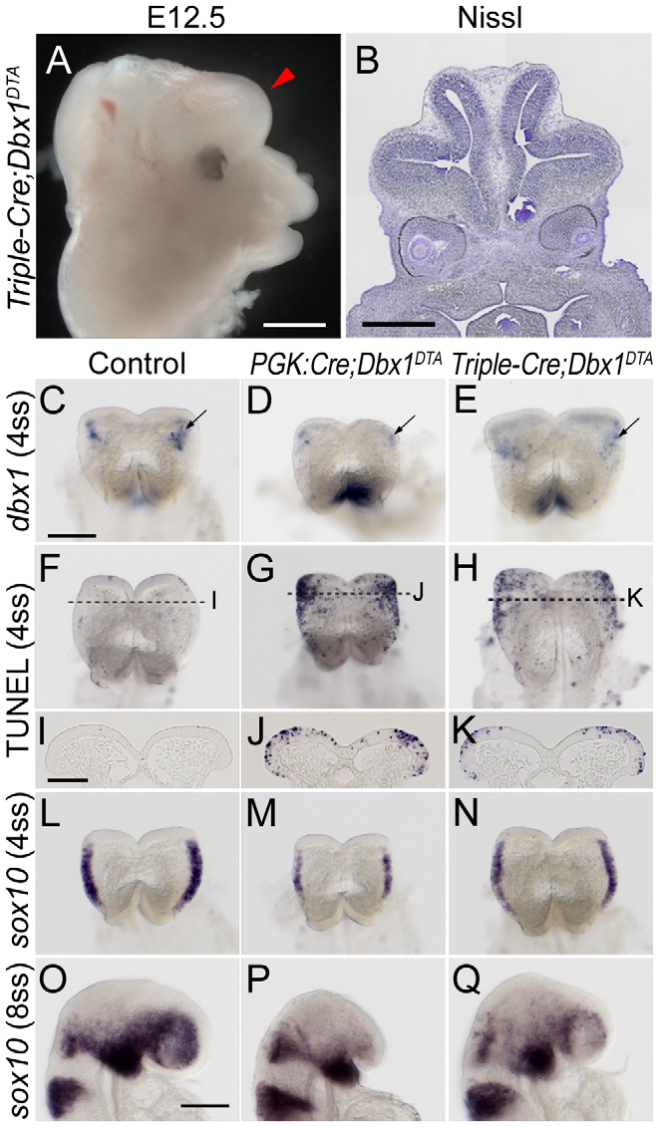
Comparison between PGK:Cre and Triple-Cre driven ablations. (A) Head of an E12.5 *Triple-Cre;Dbx1^DTA^* embryo. The red arrowhead indicates the forebrain. (B) Nissl-stained coronal section of an E12.5 *Triple-Cre;Dbx1^DTA^* embryo at the level of the eyes. (C–E) Dorsal views of whole-mount *in situ* hybridisation for *dbx1* on wild-type (C), *PGK:Cre;Dbx1^DTA^* (D), *Triple-Cre;Dbx1^DTA^* (E) 4ss embryos. A significant number of *dbx1*-expressing cells (arrows) appear to escape ablation in Triple-Cre ablated embryos. The strong staining in D corresponds to the (out of focus) rhombencephalic expression of *dbx1*, and appears obvious because revelation time had to be increased in *PGK:Cre* ablated embryos in order to visualise the very few positive cells that remain. (F–H) Whole-mount TUNEL staining on wild-type (F), *PGK:Cre;Dbx1^DTA^* (G), *Triple-Cre;Dbx1^DTA^* (H) 4ss embryos. I–K are sections of these embryos collected at the levels indicated by dashed lines. Less apoptotic cells are found in Triple-Cre than in PGK:Cre ablated embryos. (L–Q) Whole-mount *in situ* hybridisation for *sox10* on wild-type (L, O), *PGK:Cre;Dbx1^DTA^* (M, P) and *Triple-Cre;Dbx1^DTA^* (N, Q) embryos at 4ss (L–N) and 8ss (O–Q). At 4ss, *PGK:Cre;Dbx1^DTA^* embryos show a strong decrease in the number of *sox10* ^+^ premigratory CNCCs whereas *Triple-Cre;Dbx1^DTA^* are less severely affected. At 8ss, very few *sox10* ^+^ CNCCs have invaded the cephalic mesenchyme in *PGK:Cre;Dbx1^DTA^* whereas *Triple-Cre;Dbx1^DTA^* are not as severely affected. Scale bars: A, B: 500 µm; C, O: 200 µm; I: 100 µm.

To discriminate between these two possibilities, we generated *Foxg1^Cre^;Nkx2.1:Cre; Wnt1:Cre* (referred to as *Triple-Cre*) animals. We first analysed *Triple-Cre;Dbx1^DTA^* embryos at E12.5 and found that these animals exhibit craniofacial abnormalities similar to *PGK:Cre;Dbx1^DTA^* embryos (Fig. 8A): the nasal cavity was absent and the eyes were shifted medially in both mutants. However, *Triple-Cre;Dbx1^DTA^* mutants vastly differ from *PGK:Cre;Dbx1^DTA^* regarding brain development since the latter almost completely lacked a forebrain at this stage while the former formed a forebrain that was near normal in size (Fig. 8A, B). We therefore conclude that the cumulative contribution of *dbx1*-expressing cells targeted by both *PGK:Cre* and *Triple:Cre* lines (i.e. vDi, dDM, ANR and FE subsets) is required for accurate craniofacial morphogenesis. Conversely, *dbx1*-expressing cells that are targeted in *PGK:Cre* but not in *Triple-Cre* ablated mutants (i.e. belonging to the ME subset) are absolutely critical for forebrain formation.

*In situ* hybridisation for *dbx1* at 4ss confirmed that the EM subset is mostly spared in *Triple-Cre;Dbx1^DTA^* mutants (Fig. 8E). Although *dbx1* staining was decreased compared to control embryos, it was much higher than in *PGK:Cre;Dbx1^DTA^* mutants, which only display very few *dbx1* ^+^ cells (Fig. 8C–E). We then performed TUNEL staining and found that *Triple-Cre;Dbx1^DTA^* embryos display increased apoptosis at 4ss compared to controls and single ablations, but were not as severely affected as *PGK:Cre;Dbx1^DTA^* embryos (Fig. 8F–K; see also Fig. 6B–D). Consistently, 4ss *Triple-Cre* ablated mutants were more similar in size to their wild-type counterparts than to *PGK:Cre;Dbx1^DTA^* embryos (Fig. 8C–H).

Since tracing experiments showed that the EM subset generates CNCCs (Fig. 3X), we analysed CNCCs production in *Triple-Cre* and *PGK:Cre* ablated mutants by *in situ* hybridisation for *sox10*, a marker of NCCs [29]. At 4ss, when CNCCs are mainly premigratory, *PGK:Cre;Dbx1^DTA^* embryos displayed a strong reduction in *sox10* staining whereas *Triple-Cre;Dbx1^DTA^* embryos appeared significantly less affected (Fig. 8L–N). Similarly, at 8ss, when CNCCs have invaded the cephalic mesenchyme in wild-type embryos, *PGK:Cre;Dbx1^DTA^* counterparts were almost devoid of *sox10* ^+^ cells, whereas *Triple-Cre;Dbx1^DTA^* embryos were only mildly affected (Fig. 8O–Q). By contrast, all three genotypes showed similar strong staining in the branchial arch, which is mainly invaded by rhombencephalic NCCs. These results show that the severity of the forebrain phenotype, but not of the craniofacial phenotype, correlate with the loss of CNCCs.

Taken together, our results suggest that a small number of early-born *dbx1*-expressing cells and their CNCCs progeny have a unique function during early steps of forebrain development by promoting the survival and proliferation of neighbouring tissues in a non cell-autonomous manner.

## Discussion

In this study, we show that Dbx1-derived cells are essential for forebrain and craniofacial development in mice. We have identified five restricted *dbx1*-expressing subsets in the head at early (E8.25–E8.75) developmental stages (EM, vDi, dDM, ANR and FE). Complete genetic ablation of these cells results in extensive and non cell-autonomous apoptosis as well as decreased proliferation in tissues that will later give rise to the midbrain, forebrain and face, leading to dramatic defects in head morphogenesis. Careful analysis of the dynamics of *dbx1* expression in wild-types and apoptosis in ablated mutants indicated that the EM subset, which generates a small subpopulation of CNCCs, is crucial to maintain the survival of neighbouring tissues. In addition, targeted and combinatorial ablations of the different subsets, using mutants carrying multiple Cre-expressing transgenes, revealed that the vDi, dDM, ANR and FE are collectively required to ensure accurate craniofacial morphogenesis, whereas the EM is necessary for forebrain formation.

### Distinct contribution of Dbx1-derived cells to head development

*Dbx1* was previously reported to be expressed from E9.5 in the mouse brain [14], [15]. We now show that five distinct subsets of *dbx1*-expressing cells can be identified at earlier stages (E8.25–E8.75), including one in the facial ectoderm. This study is the first to describe that *dbx1* expression is not confined to the central nervous system, as previously reported [15]. Unlike other vertebrates [30] however, we did not find *dbx1* expression in the anterior axial mesendoderm of mouse embryos.

One of our most striking findings is that the presence or absence of a limited number of cells from the Dbx1 lineage can have profound consequences on brain and face morphogenesis. Thus, *PGK:Cre* and *Triple-Cre* ablated mutants, which only differ by the presence/absence of the EM subset, exhibited very similar craniofacial defects at E12.5 but vastly differed regarding their forebrain phenotype. This allowed us, therefore, to dissociate forebrain formation and facial morphogenesis, and demonstrate that these two processes rely on distinct players, namely “early” (2–4ss) and “late” (from 5–6ss) *dbx1*-expressing subsets respectively. Since all targeted ablations resulted in different craniofacial phenotypes, and none of them recapitulated the defects observed in *PGK:Cre* or *Triple-Cre* ablated mutants, our results show that each *dbx1*-expressing population has a specific role and that their coordinated activity is required to ensure accurate facial morphogenesis. Importantly, the dDM, vDi and ANR/FE *dbx1*-expressing subsets largely overlap with region producing Wnts/BMPs, Shh and Fgf8 respectively. Given the key function of these molecules in both craniofacial morphogenesis and forebrain patterning [31]–[34], they appear as good candidates to have a significant contribution to the ablation phenotype.

### Non cell-autonomous function of *dbx1*-derived cells

Apoptosis in *PGK:Cre;Dbx1^DTA^* embryos was observed from early somite stages, prior to the onset of vasculogenesis or the initiation of neural tube closure, indicating that none of these processes can account for increased cell death. Conversely, the failure of neural tube closure could arise from increased apoptosis as it is observed in several exencephalic mouse mutants [35]. The specific enrichment of apoptotic cells observed at the lateral borders of the neural plate of *PGK:Cre;Dbx1^DTA^* embryos could thus preclude fusion of the neural folds. However, the failure of forebrain maintenance in these mutants cannot be attributed to the neural tube closure defect since *Wnt1:Cre;Dbx1^DTA^* embryos, which also display an opened brain at E9.5 (data not shown), form a forebrain.

Apoptosis in *PGK:Cre;Dbx1^DTA^* embryos was observed in regions that neither express *dbx1* nor derive from *dbx1*-expressing cells and *dbx1* expression was not upregulated upon ablation suggesting that apoptosis in non cell-autonomous. We unambiguously demonstrated that cell death is indeed non cell-autonomous using targeted ablation in the presence of the *R26^YFP^* reporter allele, thus allowing us to show that a large number of apoptotic cells are not recombined in *Foxg1^Cre^* and *Wnt1:Cre* ablated mutants, and therefore cannot undergo cell death because of DTA expression. In addition to increased apoptosis, *PGK:Cre;Dbx1^DTA^* embryos also displayed a reduction in the proliferation within the neural plate. However, given the respective extent of the proliferation and apoptosis defects in *PGK:Cre;Dbx1^DTA^* embryos, we believe that the phenotype observed upon ablation mainly result from increased cell death.

We have recently shown that other later-born Dbx1-derived populations, generated between E10.5 and E12.5 and migrating over long distances to settle in the cerebral cortex, regulate proliferation and differentiation in a non cell-autonomous manner, but were not involved in regulating cell survival [17], [18]. We now bring evidence that the earliest-born Dbx1-derived cells also share the capacity to influence territories at distance from their generation site but they have a unique role in cell survival. The ability to regulate the development of adjacent tissues therefore appears to be a common feature among Dbx1-derived cells.

### Role of CNCCs during forebrain development

We have shown that the EM *dbx1*-expressing subset gives rise to a specific subpopulation of CNCCs and we have correlated the ablation of this subset with extensive apoptosis in surrounding tissues and failure in forebrain formation. Tracing experiments indicated that CNCCs are not the only progeny of the EM subset, as some EM-derived cells remain in the neural plate where they later intermingle with cells deriving from the dDM subset. Since tracing of the Dbx1 lineage and ablation are mutually exclusive strategies, it is not possible to precisely say which cells belonging to the EM subset are spared in *Triple-Cre;Dbx1^DTA^* mutants. However, the fact that (i) the *Wnt1:Cre* line drives recombination in all NCCs, (ii) apoptotic cells in 4ss *Triple-Cre;Dbx1^DTA^* mutants are mostly found in lateral regions, whereas (iii) untargeted *dbx1* ^+^ cells are more medial, suggest that Dbx1-derived CNCCs are likely to be ablated in *Triple-Cre;Dbx1^DTA^* mutants. This is also supported by the small reduction in *sox10* staining observed at 4ss following ablation with the *Triple-Cre* line, which matches the relatively small number of Dbx1-derived CNCCs found in wild-types. By contrast, the strong decrease in *sox10* staining observed in *PGK:Cre;Dbx1^DTA^* embryos suggests that a significant number of CNCCs which are not from the Dbx1 lineage undergo cell death in these mutants. It is thus likely that *dbx1*-expressing cells belonging to the EM subset control forebrain formation by regulating the production/survival of CNCCs.

To date, little is known concerning the role of CNCCs during forebrain development in mice. By contrast, this question was previously addressed using avian models. In chicken, surgical excision of the neural folds generating facial skeletogenic CNCCs prior to their delamination (2–5ss) results in the complete absence of the telencephalon [12]. When the excision is performed at slightly later stages (5–6ss), a forebrain is formed, although its patterning is severely affected and dorsal midline closure does not occur [10], [13]. Subtle variations in the timing of the ablation therefore have a profound influence on its outcome.

In our system, we mimic the consequences of a surgical removal of the mid-diencephalic to anterior-rhombencephalic neural folds, using genetic means to perform the ablation of only a very specific and molecularly characterised population of cells. In addition, we show that forebrain formation is maintained when some of the earliest *dbx1*-expressing cells are spared from the ablation.

Increased apoptosis in *PGK:Cre;Dbx1^DTA^* embryos is observed from early stages and is clearly the main cause to the phenotype we observe. In chicken experiments, increased apoptosis was observed in forebrain tissues, but it was reported 24 to 48 hours after surgery and only in the case of 2–5ss ablations [10]–[13]. The contribution of apoptosis in the avian paradigm of CNCCs depletion thus remains to be precisely assessed in order to better understand how CNCCs contribute to forebrain development.

### Mechanisms of Dbx1-derived cells function

In *PGK:Cre;Dbx1^DTA^* embryos, anterior neural territories are correctly induced but fail to be maintained and undergo extensive apoptosis. One possible explanation of our observations is that Dbx1-derived cells normally provide signals (whose identity remains to be established) necessary for cell viability or cell survival in surrounding tissues. Such a function could be fulfilled by long-range diffusing molecules originating from the neural tissue itself and/or by signals locally released by migrating CNCCs. Further studies will be required in order to discriminate between these two hypotheses. Of note, although the function of the *dbx1* gene is not precisely known during forebrain development, Dbx1 itself is most likely not involved in the processes we describe here as *dbx1*-deficient embryos form a forebrain and do not display defects in craniofacial morphogenesis ([25] and unpublished results).

A large number of mouse mutants displaying aberrant head morphogenesis have been identified [36], but in most instances the defects result from improper induction or patterning rather than increased cell death as it is the case in *PGK:Cre;Dbx1^DTA^*. A specific feature of our model is the fact that the eye fields remain unaffected despite extensive apoptosis in neighbouring areas, suggesting that the signalling partners mediating the survival of presumptive forebrain tissues are different from those involved in eye development.

All thing considered, the model that best recapitulates the phenotype of *PGK:Cre;Dbx1^DTA^* mutants is probably the CNCC ablation paradigm in chick [10], [12], [13]. However, although a mechanism involving BMP antagonists was proposed to mediate CNCCs function [11], the fact that it relies on the control of neural tube closure, forebrain growth and patterning but not on the regulation of survival suggests significant divergence between our respective systems.

In this study, we identify novel *dbx1*-expressing populations at early stages of neural development and demonstrate that, despite their limited number, they exert a critical function in maintaining forebrain and craniofacial development. In gastrulating zebrafish embryos, the ablation of only a dozen of anterior ectodermal cells was shown to result in major brain patterning defects followed by widespread apoptosis [37]. In mice, early *dbx1*-expressing cells constitute a unique example of such few cells having a crucial function during brain development.

## Supporting Information

Figure S1 **Nestin:Cre driven ablation of Dbx1-derived cells at E12.5.** Heads of wild-type (A) and *Nestin:Cre;Dbx1^DTA^* (B) E12.5 mouse embryos. The obvious reduction in the size of the midbrain in mutants is indicated by the yellow arrowhead in B. (C–D) Coronal sections collected at the level of the eyes of wild-type (C) and *Nestin:Cre;Dbx1^DTA^* (D) E12.5 mouse embryos. The red arrowhead in D points at the reduction in the thickness of the medial ganglionic eminence in mutants. Scale bars: A, C: 500 µm.
(TIF)

Figure S2 **Morphological defects in E8.5** ***PGK:Cre;Dbx1^DTA^*** **embryos.** (A) Quantification of the width of the neural plate at the level of the midbrain in 2–4ss control and *PGK:Cre;Dbx1^DTA^* embryos. (B) Quantification of the length between the pre-otic sulcus and the anterior limit of the forebrain in 3 to 8ss control and *PGK:Cre;Dbx1^DTA^* embryos. * p<0.001. (C) *In situ* hybridisation for *dbx1* on 6ss control and ablated mutants. (D) TUNEL staining of presomitic E8.0 embryos showing the normal levels of apoptosis in mutants. Embryos are shown from above (anterior is up); the red asterisk indicates the node. (E) Sections collected at the level of the spinal cord of 3ss control and *PGK:Cre;Dbx1^DTA^* embryos stained by TUNEL. Scale bars: C: 200 µm; D: 50 µm.
(TIF)

Figure S3 **Targeted ablation of the Dbx1-expressing subsets.** The *Nkx2.1:Cre* line drives recombination in a domain (arrow in A) overlapping with the vDi subset (arrow in B) and allows its ablation (C, D). The *Wnt1:Cre* line drives recombination in the dDM region (arrows in E, F) and allows the complete ablation of the *dbx1*-expressing cells in this area (G, H). Using, the *Foxg1^Cre^* line, recombination occurs in the facial ectoderm (arrowhead in I) and anterior forebrain (arrow in I) but also in a salt and pepper manner along the neural tube and mesenchyme (J). Ablation driven by the *Foxg1^Cre^* line does not significantly affect the vDi and dDM subsets (K, L). At 6ss, both the *Foxg1^Cre^* (M) and *Wnt1:Cre* (N) lines allow recombination in the mesencephalic neural plate (arrows in M and N). The *Wnt1:Cre* line also drive recombination in CNCCs (arrowhead in E and N). (O–P) Whole-mount *in situ* hybridisation for *dbx1* in control (O) and *Foxg1^Cre^;Dbx1^DTA^* (P) 14ss embryos. Arrows in O and P as well as sections (Q and S) indicate the effective ablation of the ANR subset in *Foxg1^Cre^;Dbx1^DTA^* embryos. Arrowheads in O and P as well as sections (R and T) show that the FE subset is efficiently ablated in *Foxg1^Cre^;Dbx1^DTA^* embryos. Scale bars: A: 100 µm; C, O: 200 µm; Q: 50 µm.
(TIF)

Figure S4 **Quantification of cell-autonomous and non cell-autonomous apoptosis.** Counting of the number of YFP positive/negative cells among Caspase-3^+^ cells in *FoxG1^Cre^;R26^YFP^;Dbx1^DTA^* and *Wnt1:Cre;R26^YFP^;Dbx1^DTA^* embryos. Most apoptotic cells located within the recombination domain (grey) were YFP^+^ (29/41 in the telencephalon of *FoxG1^Cre^;R26^YFP^;Dbx1^DTA^* and 116/169 in the mesencephalon and diencephalon of *Wnt1:Cre;R26^YFP^;Dbx1^DTA^* embryos). On the contrary, most Caspase-3^+^ cells found in the mesencephalon and diencephalon of *FoxG1^Cre^;R26^YFP^;Dbx1^DTA^* or in the telencephalon of *Wnt1:Cre;R26^YFP^;Dbx1^DTA^* embryos were YFP^−^ (163/185 and 69/81 respectively), ruling out the possibility that these cells died because of DTA expression.
(TIF)

Figure S5 **Patterning defects in Wnt1:Cre ablated embryos.** Side (A, C) and front (B, D) views of *foxg1* expression in control (A, B) and *Wnt1:Cre;Dbx1^DTA^* (C, D) 8ss embryos. The arrows in C and D point to the dorsal and medial expansion of the expression domain respectively. Side (E, G) and top (F, H) views of *emx2* expression in control (E, F) and *Wnt1:Cre;Dbx1^DTA^* (G, H) 9ss embryos. Ablated mutants display a posterior shift in the dorsal limit of *emx2* expression (arrows). Scale bar: 200 µm.
(TIF)
