# Supplementary figures and images for: Dbx1-Expressing Cells Are Necessary for the Survival of the Mammalian Anterior Neural and Craniofacial Structures
Source: PLoS One. 2011 Apr 28;6(4):e19367. doi: 10.1371/journal.pone.0019367 (PMC3084286; doi:10.1371/journal.pone.0019367)

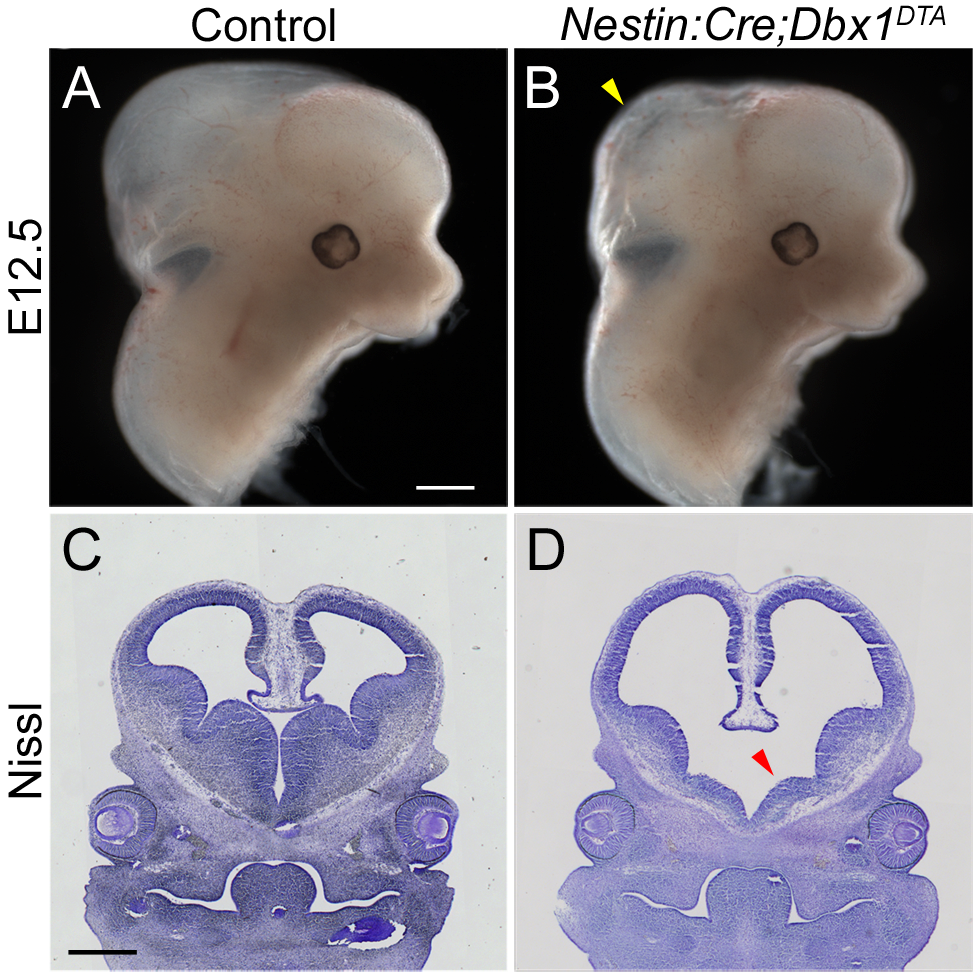

Supplement: Figure S1 — Nestin:Cre driven ablation of Dbx1-derived cells at E12.5. Heads of wild-type (A) and Nestin:Cre;Dbx1DTA (B) E12.5 mouse embryos. The obvious reduction in the size of the midbrain in mutants is indicated by the yellow arrowhead in B. (C–D) Coronal sections collected at the level of the eyes of wild-type (C) and Nestin:Cre;Dbx1DTA (D) E12.5 mouse embryos. The red arrowhead in D points at the reduction in the thickness of the medial ganglionic eminence in mutants. Scale bars: A, C: 500 µm. (TIF) [file pone.0019367.s001.tif]

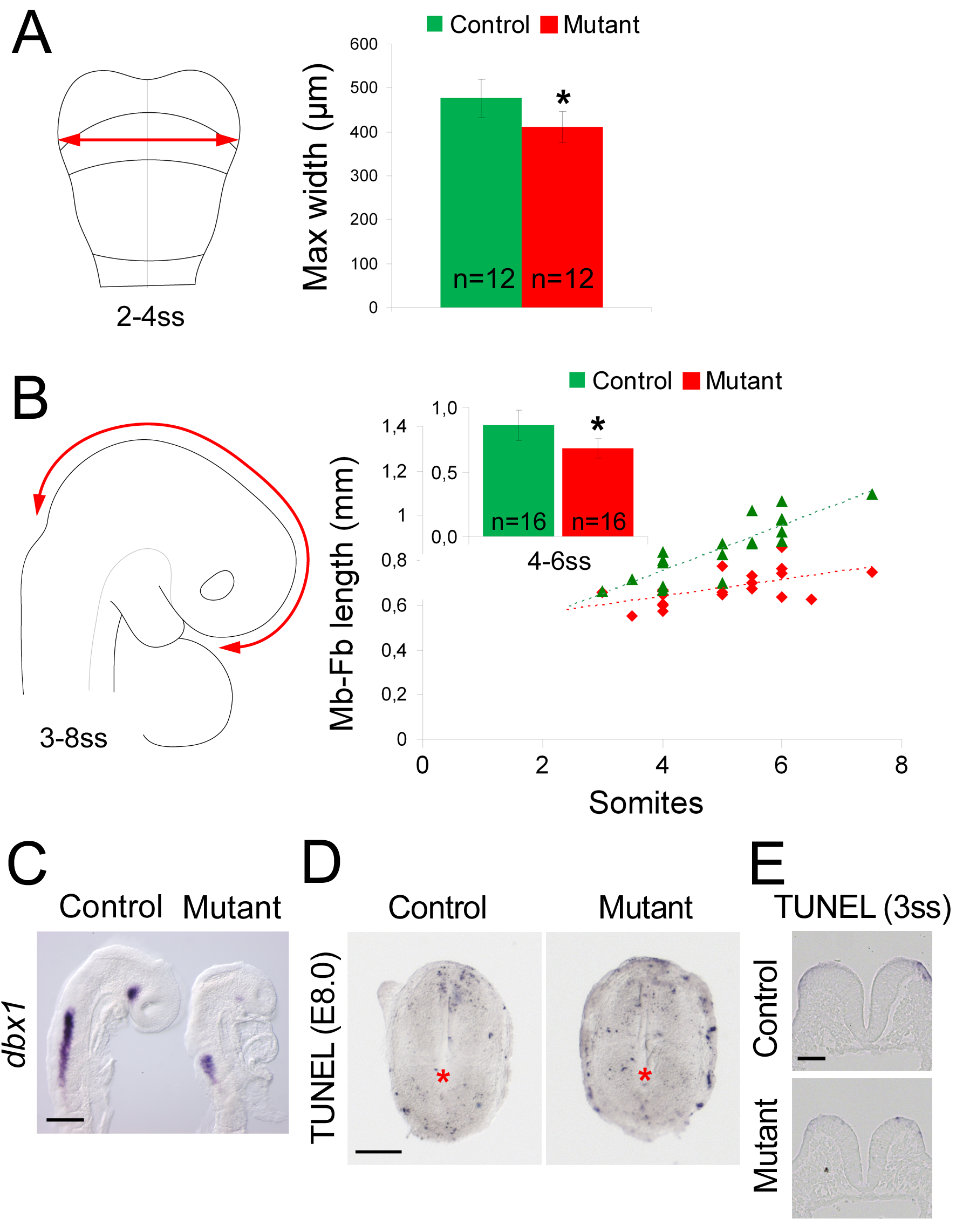

Supplement: Figure S2 — Morphological defects in E8.5 PGK:Cre;Dbx1DTA embryos. (A) Quantification of the width of the neural plate at the level of the midbrain in 2–4ss control and PGK:Cre;Dbx1DTA embryos. (B) Quantification of the length between the pre-otic sulcus and the anterior limit of the forebrain in 3 to 8ss control and PGK:Cre;Dbx1DTA embryos. * p<0.001. (C) In situ hybridisation for dbx1 on 6ss control and ablated mutants. (D) TUNEL staining of presomitic E8.0 embryos showing the normal levels of apoptosis in mutants. Embryos are shown from above (anterior is up); the red asterisk indicates the node. (E) Sections collected at the level of the spinal cord of 3ss control and PGK:Cre;Dbx1DTA embryos stained by TUNEL. Scale bars: C: 200 µm; D: 50 µm. (TIF) [file pone.0019367.s002.tif]

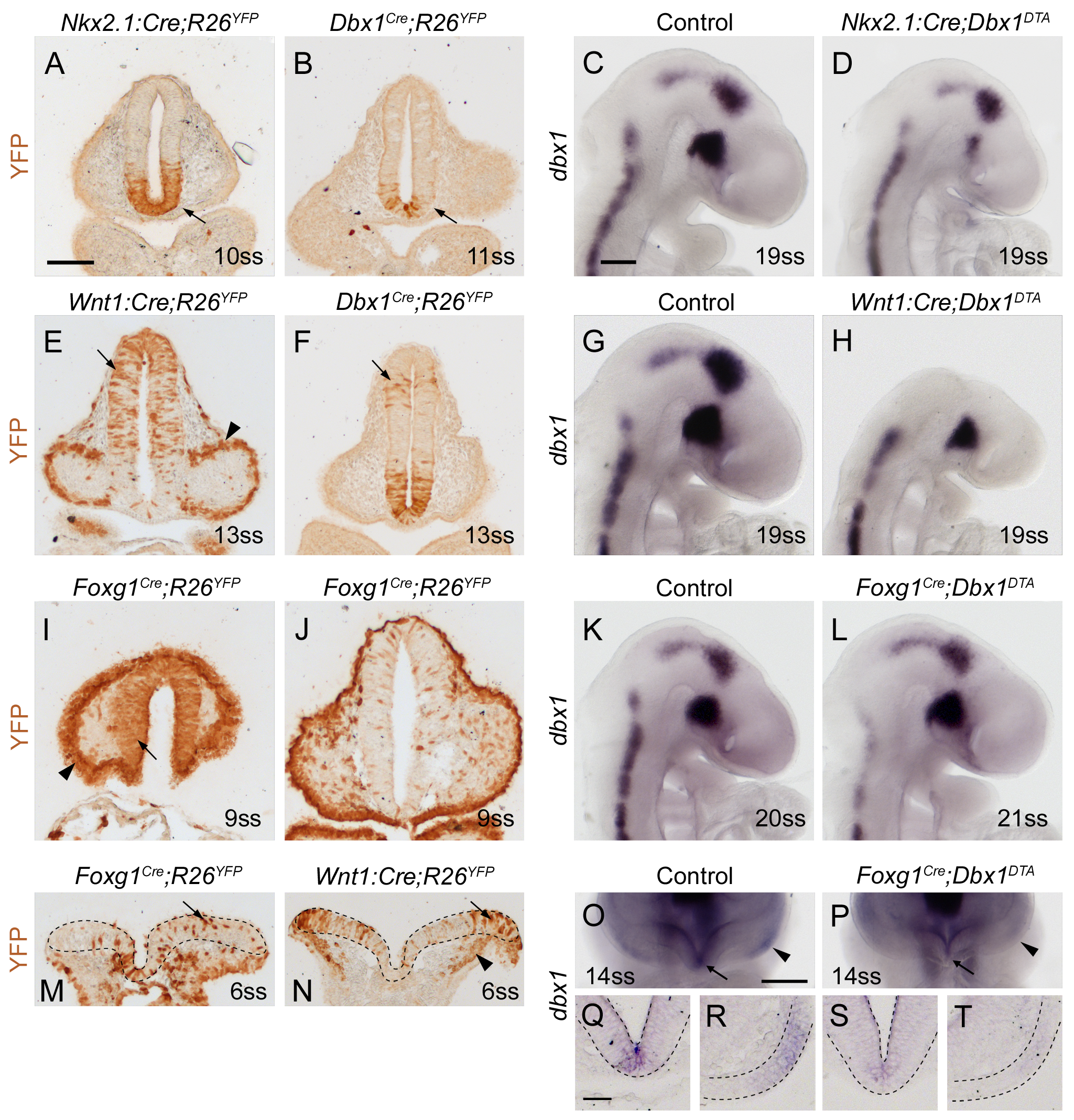

Supplement: Figure S3 — Targeted ablation of the Dbx1-expressing subsets. The Nkx2.1:Cre line drives recombination in a domain (arrow in A) overlapping with the vDi subset (arrow in B) and allows its ablation (C, D). The Wnt1:Cre line drives recombination in the dDM region (arrows in E, F) and allows the complete ablation of the dbx1-expressing cells in this area (G, H). Using, the Foxg1Cre line, recombination occurs in the facial ectoderm (arrowhead in I) and anterior forebrain (arrow in I) but also in a salt and pepper manner along the neural tube and mesenchyme (J). Ablation driven by the Foxg1Cre line does not significantly affect the vDi and dDM subsets (K, L). At 6ss, both the Foxg1Cre (M) and Wnt1:Cre (N) lines allow recombination in the mesencephalic neural plate (arrows in M and N). The Wnt1:Cre line also drive recombination in CNCCs (arrowhead in E and N). (O–P) Whole-mount in situ hybridisation for dbx1 in control (O) and Foxg1Cre;Dbx1DTA (P) 14ss embryos. Arrows in O and P as well as sections (Q and S) indicate the effective ablation of the ANR subset in Foxg1Cre;Dbx1DTA embryos. Arrowheads in O and P as well as sections (R and T) show that the FE subset is efficiently ablated in Foxg1Cre;Dbx1DTA embryos. Scale bars: A: 100 µm; C, O: 200 µm; Q: 50 µm. (TIF) [file pone.0019367.s003.tif]

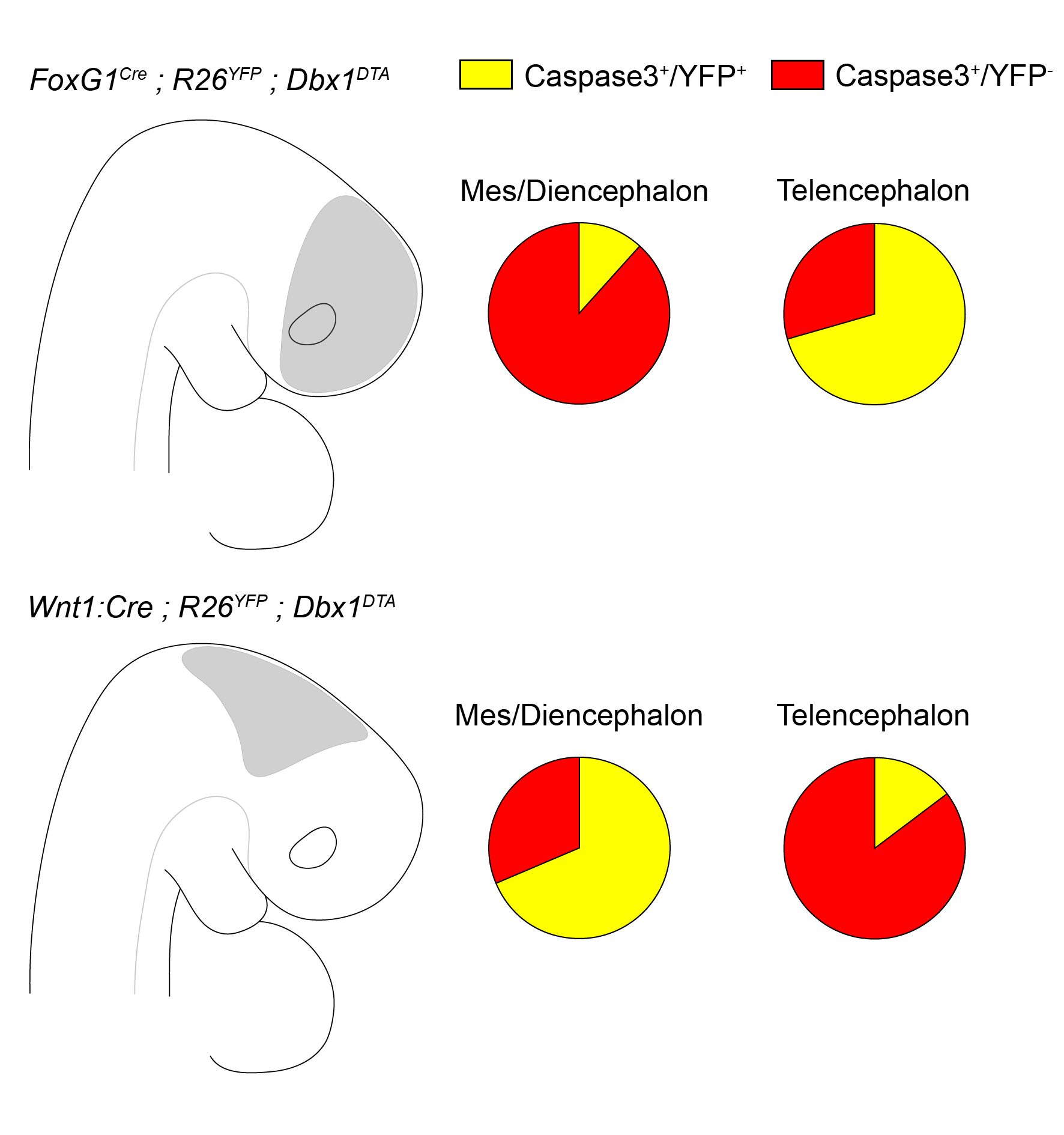

Supplement: Figure S4 — Quantification of cell-autonomous and non cell-autonomous apoptosis. Counting of the number of YFP positive/negative cells among Caspase-3+ cells in FoxG1Cre;R26YFP;Dbx1DTA and Wnt1:Cre;R26YFP;Dbx1DTA embryos. Most apoptotic cells located within the recombination domain (grey) were YFP+ (29/41 in the telencephalon of FoxG1Cre;R26YFP;Dbx1DTA and 116/169 in the mesencephalon and diencephalon of Wnt1:Cre;R26YFP;Dbx1DTA embryos). On the contrary, most Caspase-3+ cells found in the mesencephalon and diencephalon of FoxG1Cre;R26YFP;Dbx1DTA or in the telencephalon of Wnt1:Cre;R26YFP;Dbx1DTA embryos were YFP− (163/185 and 69/81 respectively), ruling out the possibility that these cells died because of DTA expression. (TIF) [file pone.0019367.s004.tif]

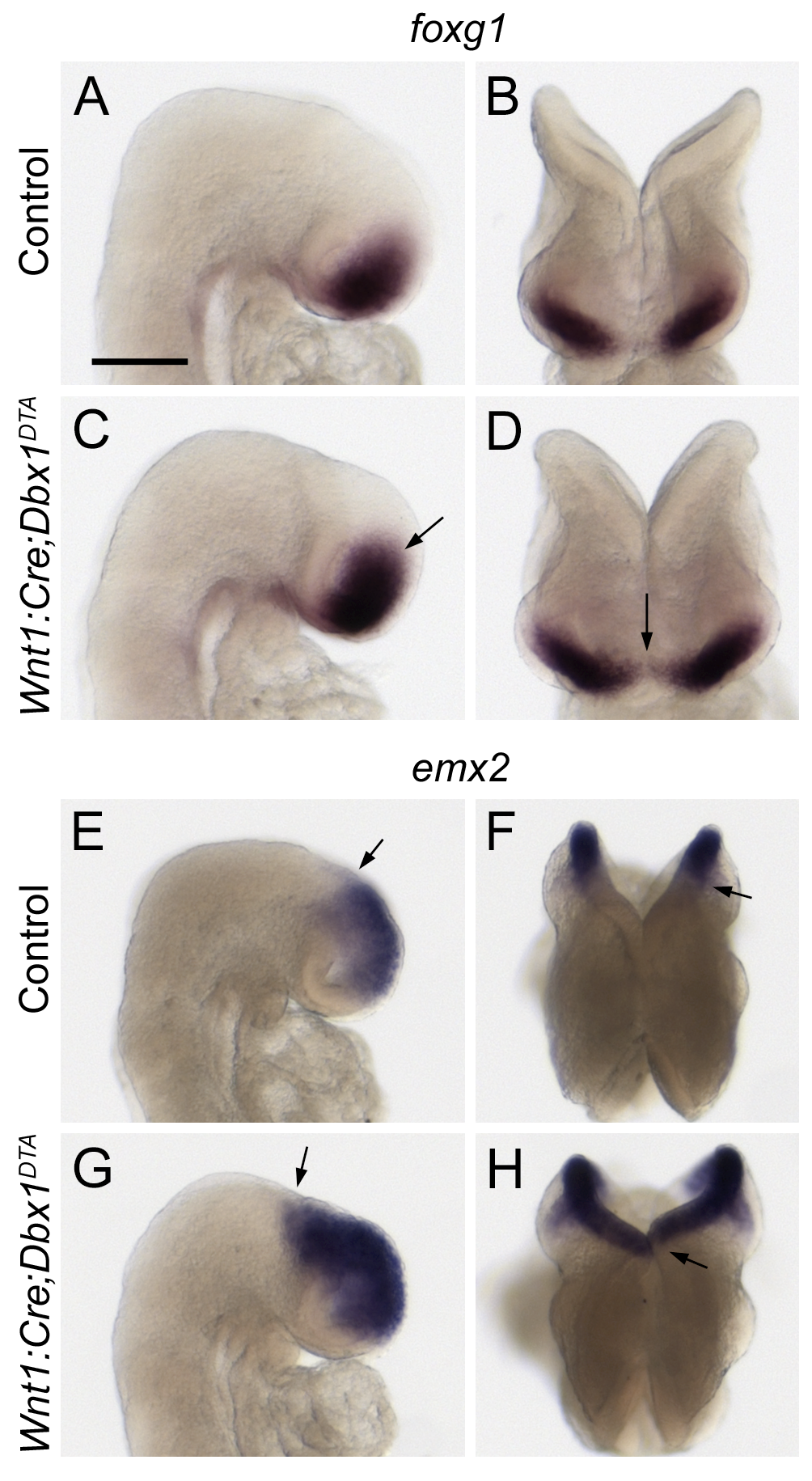

Supplement: Figure S5 — Patterning defects in Wnt1:Cre ablated embryos. Side (A, C) and front (B, D) views of foxg1 expression in control (A, B) and Wnt1:Cre;Dbx1DTA (C, D) 8ss embryos. The arrows in C and D point to the dorsal and medial expansion of the expression domain respectively. Side (E, G) and top (F, H) views of emx2 expression in control (E, F) and Wnt1:Cre;Dbx1DTA (G, H) 9ss embryos. Ablated mutants display a posterior shift in the dorsal limit of emx2 expression (arrows). Scale bar: 200 µm. (TIF) [file pone.0019367.s005.tif]
